# Immediate post-breakfast physical activity improves interstitial postprandial glycemia: a comparison of different activity-meal timings

**DOI:** 10.1007/s00424-019-02300-4

**Published:** 2019-08-08

**Authors:** Thomas P. J. Solomon, Eloise Tarry, Chloe O. Hudson, Alice I. Fitt, Matthew J. Laye

**Affiliations:** 1 School of Sport, Exercise, and Rehabilitation Sciences, College of Life and Environmental Sciences, University of Birmingham, Edgbaston, B15 2TT UK; 2 Institute of Metabolism and Systems Research, College of Medical and Dental Sciences, University of Birmingham, Edgbaston, UK; 3 Department of Health and Human Performance, College of Idaho, Caldwell, ID USA

**Keywords:** Exercise-meal timing, Exercise, Glycemic control, Walking, Standing, Circuit training, CGM, Continuous glucose monitoring, Glycemic variability, Inactivity, Sitting time, Sitting breaks, Office workers

## Abstract

The optimal timing between meal ingestion and simple physical activity for improving blood glucose control is unknown. This study compared the effects of physical activity on postprandial interstitial glucose responses when the activity was conducted either immediately before, immediately after, or 30 min after breakfast. Forty-eight adults were randomized to three separate physical activity interventions: standing still (for 30 min), walking (for 30 min), and bodyweight exercises (3 sets of 10 squats, 10 push-ups, 10 lunges, 10 sit-ups). In each intervention, 16 participants completed four trials (A to D) during which a 500 kcal mixed nutrient liquid breakfast meal was consumed. Interstitial glucose responses were recorded using continuous glucose monitoring for 2 h after the meal. The activity was completed either after the glucose monitoring period (trial A; control) or immediately before (trial B), immediately after (trial C), or 30 min after (trial D) the breakfast. Mean, coefficient of variance (CV), and area under the curve (AUC) for glucose were calculated and compared between the four trials. Walking and bodyweight exercises immediately after the meal improved mean, CV, and AUC glucose (*P* ≤ 0.05 vs. control), while standing immediately after the meal only improved AUC glucose (*P* ≤ 0.05 vs. control) and nearly improved mean glucose (*P* = 0.06). Mean, CV, and AUC glucose were not affected by standing, walking, or bodyweight exercise conducted immediately before, or 30 min after the meal (all *P* > 0.05 vs. control). Energy intake (diet records) and energy expenditure (Actigraph) were consistent throughout the studies and did not influence the findings. Low- to moderate-intensity activity should be implemented soon after eating to improve glucose control following breakfast. The type of activity appears less important than the timing. These findings will help optimize exercise-meal timing in general health guidelines. ClinicalTrials.gov Identifier: NCT03730727

**Electronic supplementary material:**

The online version of this article (10.1007/s00424-019-02300-4) contains supplementary material, which is available to authorized users.

## Introduction

Physical inactivity is associated with poor blood glucose control and increased incidence of type 2 diabetes and cardiovascular disease [5, 17, 18, 25, 32]. Adults are advised to accumulate ≥ 150 min/week of moderate-intensity activity [2, 20, 44]. These activity guidelines are also included in diabetes prevention strategies [2]. Type 2 diabetes is characterized by elevated HbA1c that increases the risk of cardiovascular-related mortality. Persistent postprandial hyperglycemia is the predominant contributor to HbA1c and is also associated with cardiovascular disease risk in people with and without diabetes [10, 31]. Because people spend a large part of the day in a postprandial state [16], which is often sedentary time, management of postprandial hyperglycemia is highly relevant even in individuals without diabetes. Furthermore, in healthy individuals, elevated blood glucose levels are still associated with increased inflammation and endothelial dysfunction [15, 30]. However, health guidelines do not specify when activity should be done to best optimize postprandial hyperglycemia.

Since 2001, some studies [8, 12, 23, 33, 34, 36, 41, 43] have highlighted the potential importance of activity-meal timing in relation to blood glucose control (reviewed in [39]). However, the number of studies is sparse and the sample sizes are small. Some studies have no control [41] or pre-meal activity group [36], and some are retrospective diet and exercise log analyses [34, 41]. Other studies have used either long duration (2 h) [8] or vigorous [23, 41, 43] activities that are effective in reducing postprandial glucose but not always feasible in the real-world. Consequently, outcomes from these studies are equivocal. In 2014, a viewpoint was published stating that mid-postprandial moderate-intensity activity (30–120-min post-ingestion) would best optimize postprandial hyperglycemia [11]. The viewpoint was based on anecdotes from the author’s self-management of hyperglycemia, and although it was not experimental, it underlined a very important knowledge gap. However, as of 2019, clarity concerning the optimal activity-meal timing for improving postprandial glycemia is still required.

Examining the acute effect of activity is helpful for understanding the daily regulation of glucose control. This has relevance to preventing hyperglycemia in non-diabetic individuals and to managing diabetes, where the goal is to rapidly optimize HbA1c by reducing postprandial hyperglycemia and glycemic variability while avoiding hypoglycemia [3]. To help improve current recommendations for optimizing postprandial glucose, this study compared the effects of physical activity on postprandial interstitial glucose responses when the activity was conducted either immediately before, immediately after, or 30 min after breakfast. Since it is important to consider the type of activity that can feasibly be integrated into a lifestyle change, parallel studies examined three different types of low- to moderate-intensity activities that raise energy expenditure: standing, walking, and bodyweight exercises.

## Methods

### Subjects

**Fig. 1:**
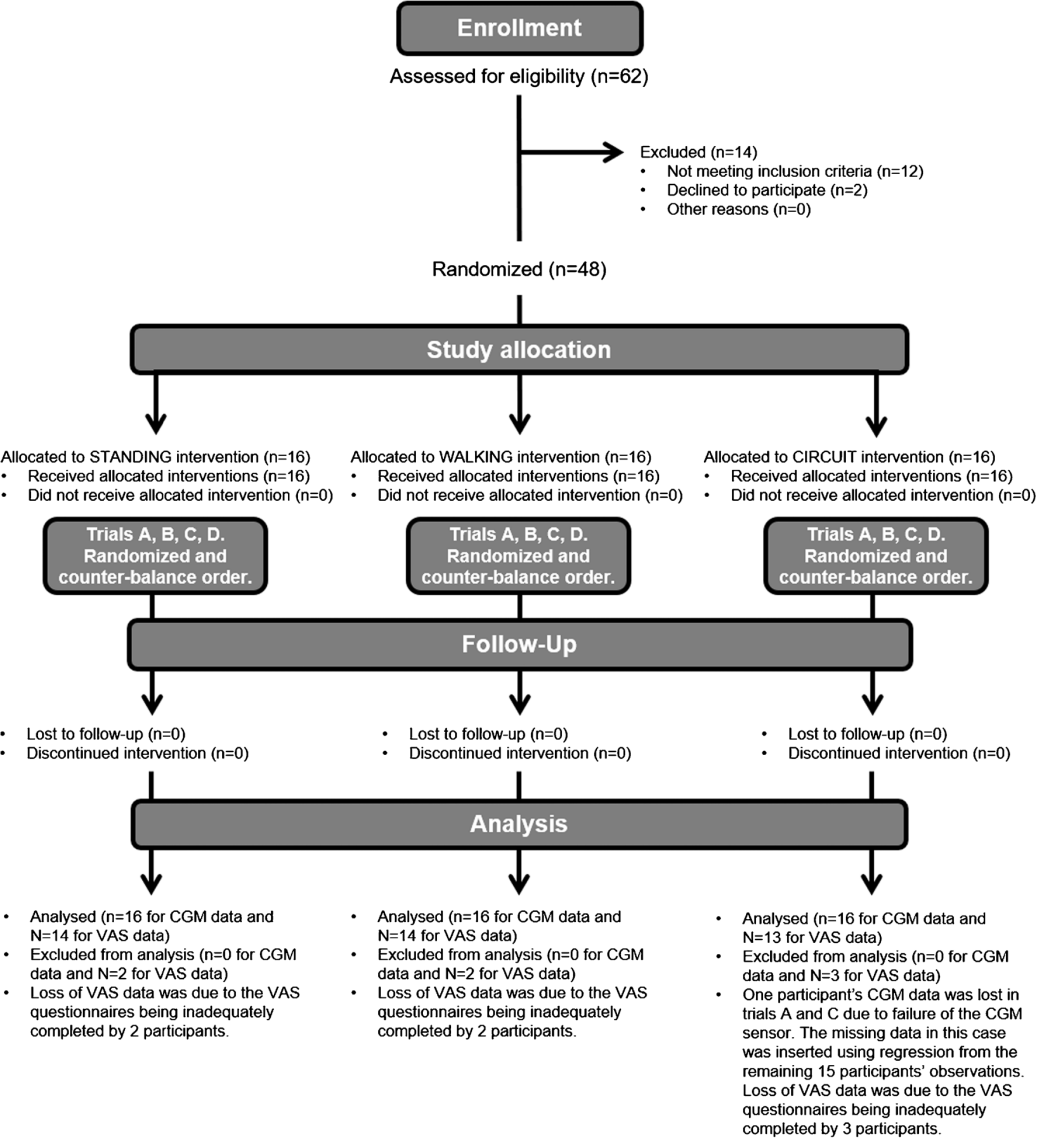
Study design. CONSORT diagram explaining the study design. Forty-eight participants were randomized to one of three studies. Each study involved a different type of activity: standing still for 30 min, treadmill walking for 30 min at a self-selected brisk pace, or 3 sets of 10 squats, 10 push-ups, 10 sit-ups, and 20 alternate leg forward lunges. In each study, participants completed four experimental trials, A to D, in a randomized, counter-balanced order. Trial A—control—participants ingested breakfast and completed the physical activity bout at some point during the rest of the day. Trial B—pre-meal activity—participants completed the physical activity bout then immediately ingested breakfast. Trial C—immediate post-meal activity—participants ingested breakfast then immediately completed the physical activity bout. Trial D—delayed post-meal activity—participants ingested breakfast then completed the physical activity bout 30 min later

The study was ethically approved (ERN_18-0942) and registered (NCT03730727). Power calculations determined that 14 participants would achieve 80% power to detect differences at the 5% level. To account for attrition, 16 participants were recruited to each of the three parallel studies, which began 10/2017 and finished 11/2018 (Fig. 1). Participants provided informed consent before medical screening and were included if they were aged 18–65 years old with a BMI 18–30 kg/m^2^, generally healthy and physically active, and excluded if they smoked, were pregnant, had signs or symptoms of chronic disease, or contraindications to exercise.

### Study design

Forty-eight participants (Table 1) were randomized to one of three studies (Fig. 1) involving a different type of activity: Standing still for 30 min, treadmill walking for 30 min at a self-selected brisk pace, or 3 sets of 10 squats, 10 push-ups, 10 sit-ups, 20 alternate leg forward lunges. In each study, participants visited the lab on five consecutive days for a pre-trial visit and four experimental trials. The order of experimental trials was randomized (http://www.randomization.com/). At the pre-trial visit, participants were given diet records and instructed to maintain their typical diet and activity habits. Participants were then fitted with a heart rate-enabled waist-worn tri-axial accelerometer (Actigraph-wGT3X-BT, Pensacola, FL) to measure daily energy expenditure and sedentary time, and a continuous glucose monitor (CGM; Dexcom G5 mobile, Camberley, UK), to wear for the following 4 days. At the end the pre-trial visit, participants completed the bout of physical activity to be performed on the trial days and then returned to the lab the following morning for their first experimental trial:

- Trial A—control—participants arrived at the laboratory at 8 a.m. following an 8–10-h overnight fast. A 500-kcal breakfast (meal-replacement drink; Nurishment, Dunn’s River, USA) containing 71 g of carbohydrate (57% total kcal), 13 g of fat (26% total kcal), and 24 g of protein (19% total kcal) was then provided. Participants were instructed to finish the meal within 10 min. After the 2-h postprandial measurement period had finished, participants completed the physical activity bout at some point during the rest of the day.
- Trial B—pre-meal activity—identical to trial A except that the activity was completed at 8 a.m. and breakfast was ingested immediately after. Two hours after ingesting breakfast, participants left the lab.
- Trial C—immediate post-meal activity—identical to trial A except that the activity was completed immediately after breakfast was ingested.
- Trial D—delayed post-meal activity—identical to trial A except that the activity was completed 30 min after breakfast was ingested.

**Table 1:** Subject characteristics

Subject characteristics	Standing	Walking	Bodyweight exercises
*N*	16	16	16
Sex	11 ♂, 5 ♀	5 ♂, 11 ♀	9 ♂, 7 ♀
Ethnicity	16 Caucasian	13 Caucasian 2 African 1 Asian	13 Caucasian 1 African 2 Asian
Age (year)	31 ± 11	24 ± 7	29 ± 12
Weight (kg)	72.3 ± 10.6	66.0 ± 11.3	65.8 ± 8.9
BMI (kg/m^2^)	23.5 ± 2.8	23.0 ± 3.4	23.0 ± 2.8
Waist circumference (cm)	82.1 ± 7.4	79.0 ± 7.4	77.6 ± 5.5
Resting heart rate (bpm)	59 ± 13	68 ± 12	56 ± 13
Resting systolic BP (mmHg)	125 ± 8	116 ± 10	125 ± 6
Resting diastolic BP (mmHg)	81 ± 8	73 ± 6	77 ± 7
HbA1c (%)	5.3 ± 0.3	5.1 ± 0.4	5.1 ± 0.3
HbA1c (mmol/mol)	34.2 ± 3.5	32.5 ± 4.6	32.0 ± 3.2
Fasting glucose (mM)	5.22 ± 0.62	5.00 ± 0.33	4.89 ± 0.36
Fasting triglycerides (mM)	1.21 ± 0.36	1.37 ± 0.54	1.07 ± 0.20
Fasting cholesterol (mM)	4.12 ± 0.48	4.50 ± 0.64	4.51 ± 0.41
Daily physical activity (kcals/day)	439 ± 291	701 ± 444	294 ± 283
Daily moderate to vigorous physical activity (min)	54 ± 37	92 ± 35	41 ± 38
Daily step counts (steps/day)	7985 ± 4686	13,008 ± 3839	6042 ± 5198
Daily sedentary time (min/day)	660 ± 305	755 ± 160	342 ± 224
Daily energy intake (kcal/day)	2110 ± 511	1972 ± 452	1959 ± 203
Daily carbohydrate intake (% of kcal)	50 ± 5	48 ± 4	49 ± 5
Daily fat intake (kcal)	33 ± 5	34 ± 5	33 ± 5
Daily protein intake (kcal)	18 ± 2	18 ± 4	18 ± 3

Data represent mean ± SD from the three groups of participants who completed each study. Age, sex, resting heart rate, blood pressure, height, weight, and waist circumference were measured by standard techniques during medical screening. Glycated hemoglobin (Radiometer Hemocue HbA1c 501, Copenhagen, Denmark), cholesterol, and triglycerides (Roche Diagnostics Accutrend Plus, Burgess Hill, UK) were measured in capillary blood samples and also collected during the screening visit

Heart rate, blood pressure, and ratings of perceived exertion (RPE; 20-point Borg scale) were recorded at the end of each activity bout. Participants also completed a visual analogue scale (VAS; 10 cm) questionnaire evaluating gastrointestinal sensations. Using indirect calorimetry (Viasys Vmax, Yorba Linda, CA), total energy expenditure and respiratory exchange ratios (RER) were measured during the physical activity bouts in 12 participants who undertook each of the three activities (walking, standing, and bodyweight exercises) on separate days.

### Calculations

Postprandial interstitial glucose concentrations were measured for 2 h after each meal. Mean glucose and the area under the glucose response curve (AUC) during the 2 h, indices of “glucose exposure” [13], were calculated. The coefficient of variance (CV) of glucose during the 2-h, an index of “glucose variability” [13], was also calculated. Accelerometry data were analyzed using Actilife (ActigraphCorp, Pensacola, FL). Diet records were analyzed using the UK food database installed in My Fitness Pal (Under Armor, Baltimore, MD).

### Statistics

Prism v7 (GraphPad, La Jolla, CA, USA) was used to perform all analyses. Normality and homogeneity of variance tests were applied, and variables diverging from parametric assumptions were log-transformed prior to analysis. The effects of the trials were compared within the three studies (standing, walking, and bodyweight exercises) using three-way ANOVA. Since it was not an a priori aim to statistically compare the three types of activity, each type was analyzed independently. Glucose time-course responses between trials were compared using a two-way ANOVA. Mean, %CV, and AUC glucose were compared between trials using one-way ANOVA. Between-trial and pre-/post-activity differences in visual analogue scale questionnaires were analyzed using two-way ANOVA. Between-trial differences in caloric intake, macronutrient composition, and physical activity were compared within each study using one-way ANOVA. Tukey post hoc tests were applied. Sex was used as a covariate but did not influence any outcome measure. Statistical significance was accepted when *P* ≤ 0.05. All data represent mean ± SD.

## Results

### Standing

**Fig. 2:**
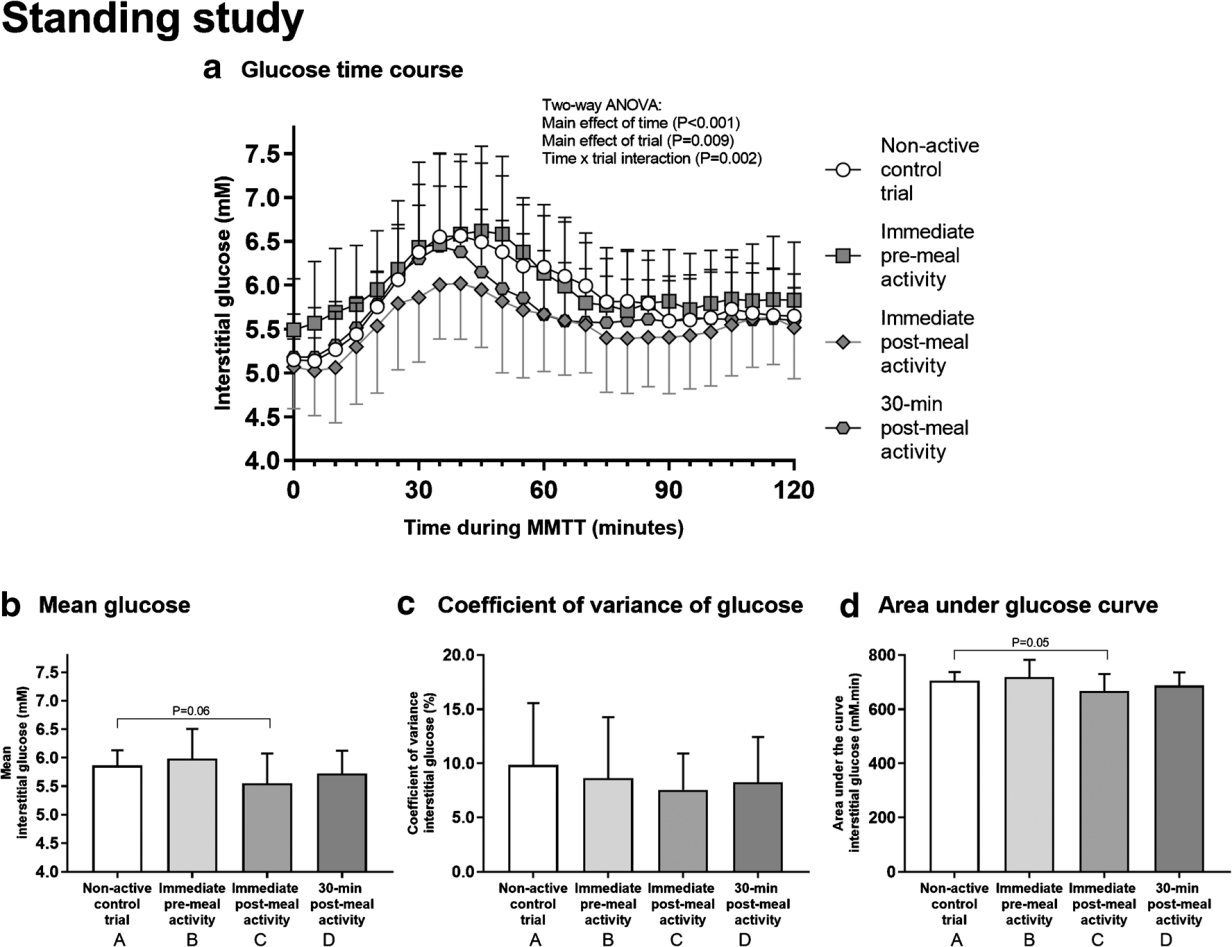
The effect of standing on glucose control. During each trial, a 500-kcal breakfast meal was provided. The trials were identical except that participants stood for 30 min either immediately before (trial B), immediately after (trial C), 30 min after (trial D), or more than 2 h after (control; trial A) meal ingestion. Interstitial glucose concentrations were measured for 2 h postprandially (**a**). The mean glucose (**b**), coefficient of variation of glucose (CV; **c**), and area under the glucose curve (AUC; **d**) during the 2-h postprandial period were calculated. Two-way ANOVA showed a significant main effect of time (*P* < 0.001), trial (*P* = 0.009), and a time × trial interaction (*P* = 0.002) for glucose time-course responses to meal ingestion (**a**). One-way ANOVA showed a main effect of trial for mean glucose (*P* < 0.05; **b**), but post hoc tests did not identify significant differences between trials (all comparisons *P* > 0.05 including *P* = 0.06 for immediate post-meal standing vs. control). The main effect of trial for CV was not significant but likely underpowered (*P* = 0.06; **c**). A main effect of trial was found for AUC (*P* < 0.05; **d**); post hoc tests showed a significant difference between immediate post-meal standing and control (*P* = 0.05). Data represent mean ± SD from *N* = 16 participants

All participants stood still with minimal fidgeting for 30 min in all trials. Mean RPE (6 ± 1 arbitrary units [au]) during standing was not different between trials (*P* = 0.33). Total energy expenditure during the stand was 55 ± 14 kcal, and RER was 0.87 ± 0.10 au. The postprandial interstitial glucose time-course responses to meal ingestion are shown in Fig. 2a. Between-trial comparisons for mean glucose did not identify significant differences between trials (Fig. 2b; all comparisons *P* > 0.05), but immediate post-meal standing vs. control including yielded a *P* value of 0.06. Glucose CV also showed near-significant main effect of trial (*P* = 0.06; Fig. 2c), while glucose AUC was significantly different between the immediate post-meal standing and control trials (*P* = 0.05; Fig. 2d). None of the measures of daily physical activity were different between trials (data not presented). Although daily energy intake was different between control and immediate pre-meal standing (1698 ± 554 vs. 2131 ± 666 kcal, *P* < 0.05), when used as a covariate, daily energy intake did not influence between-trial differences in glucose concentrations (data not presented).

### Walking

**Fig. 3:**
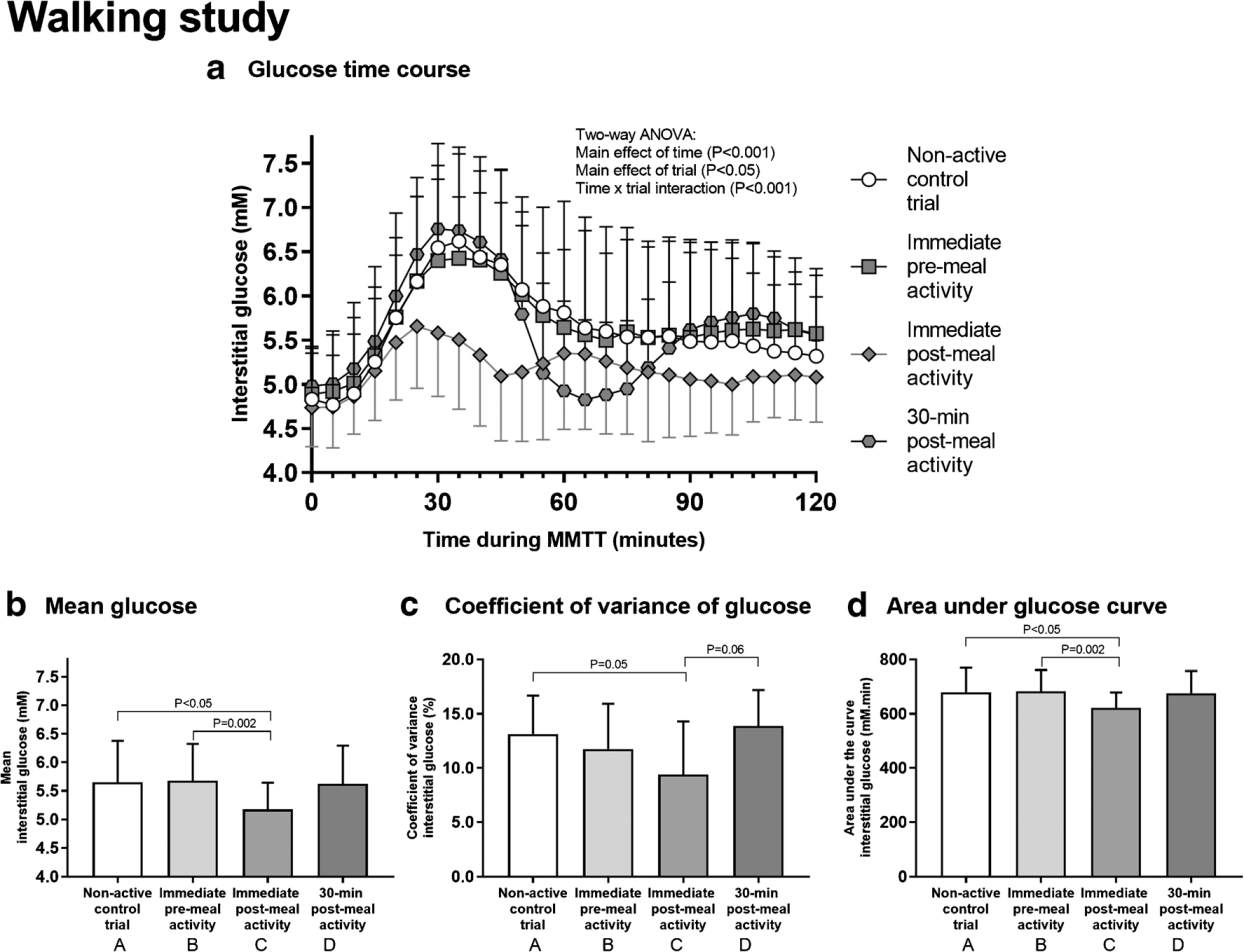
The effect of walking on glucose control. During each trial, a 500-kcal breakfast meal was provided. The trials were identical except that participants walked for 30 min either immediately before (trial B), immediately after (trial C), 30 min after (trial D), or more than 2 h after (control; trial A) meal ingestion. Interstitial glucose concentrations were measured for 2 h postprandially (**a**). The mean glucose (**b**), coefficient of variation of glucose (CV; **c**), and area under the glucose curve (AUC; **d**) during the 2-h postprandial period were calculated. Two-way ANOVA showed a significant main effect of time (*P* < 0.001), trial (*P* < 0.05), and a time × trial interaction (*P* < 0.001) for glucose time-course responses to meal ingestion (**a**). One-way ANOVA showed a main effect of trial for mean glucose (*P* < 0.05; **b**), with post hoc tests revealing that immediate post-meal walking was significantly different to control (*P* < 0.05) and pre-meal walking (*P* = 0.002). There was also a main effect of trial for CV (*P* = 0.01; **c**), with post hoc tests revealing that immediate post-meal walking was significantly different to control (*P* = 0.05). A main effect of trial was also found for AUC (*P* < 0.05; **d**), with post hoc tests showing that immediate post-meal walking was significantly different to control (*P* < 0.05) and pre-meal walking (*P* = 0.002). Data represent mean ± SD from *N* = 16 participants

All participants walked for 30 min in all trials at a self-selected speed of 3.5 ± 0.5 mph. Mean RPE (11 ± 2 au) during walking was not different between trials (*P* = 0.88). Total energy expenditure during the walk was 123 ± 19 kcal, and RER was 0.87 ± 0.06 au. The postprandial interstitial glucose time-course responses to meal ingestion are presented in Fig. 3a. Mean glucose in the immediate post-meal walking trial was significantly different to control (*P* < 0.05; Fig. 3b) and pre-meal walking (*P* = 0.002; Fig. 3b). Glucose CV in the immediate post-meal walking trial was significantly different to control (*P* = 0.05; Fig. 3c). Glucose AUC in the immediate post-meal walking trial was significantly different to control (*P* < 0.05; Fig. 3d) and pre-meal walking (*P* = 0.002; Fig. 3d). Measures of daily physical activity and daily energy intake were not different between trials (data not presented), except for sedentary time which differed between control and pre-meal walking (544 ± 229 vs. 750 ± 202 min; *P* = 0.005). However, when used as a covariate, sedentary time did not influence between-trial differences in glucose concentrations (data not presented).

### Bodyweight exercises

**Fig. 4:**
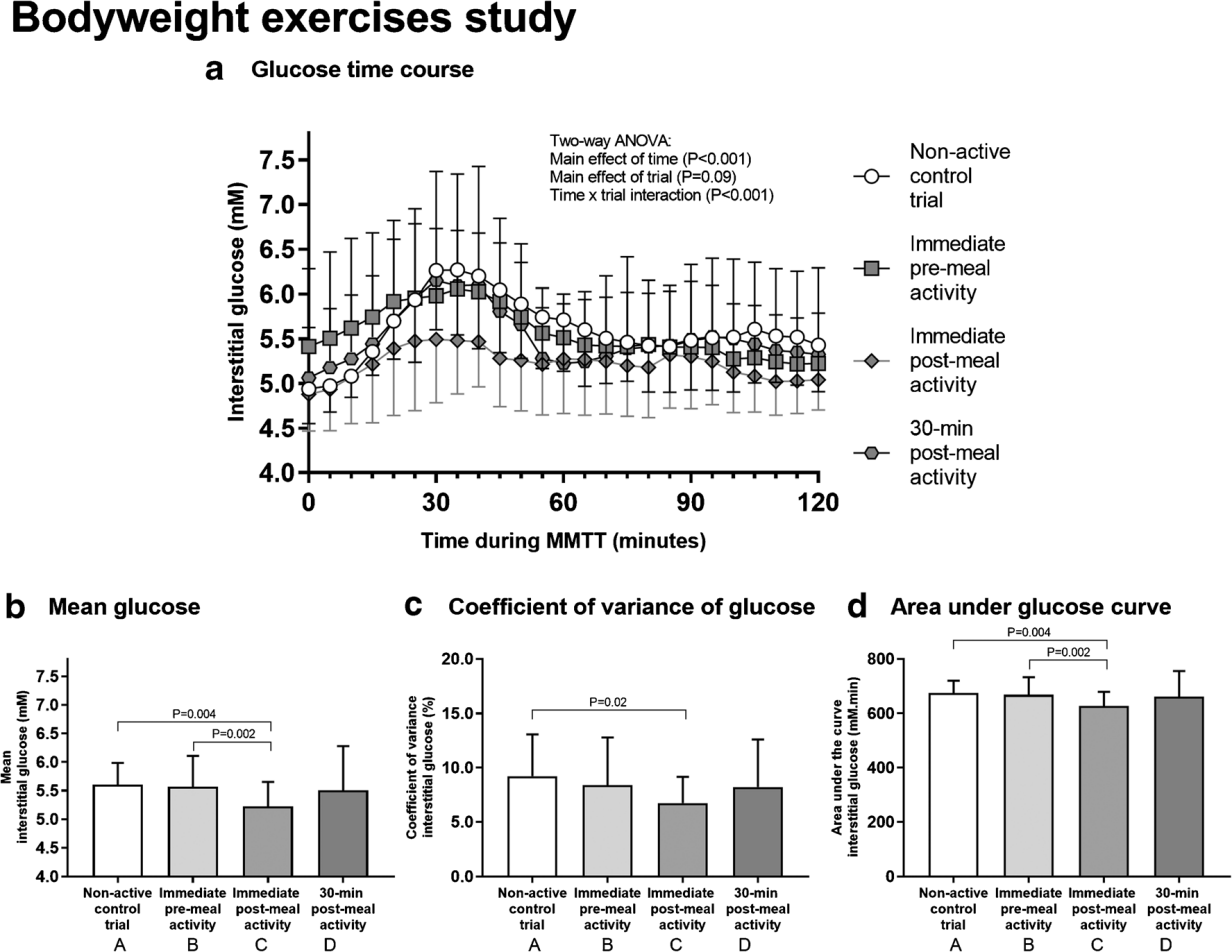
The effect of bodyweight exercise on glucose control. During each trial, a 500-kcal breakfast meal was provided. The trials were identical except that participants completed a bout of bodyweight exercises (3 sets of 10 squats, push-ups, lunges, and sit-ups) either immediately before (trial B), immediately after (trial C), 30 min after (trial D), or more than 2 h after (control; trial A) meal ingestion. Interstitial glucose concentrations were measured for 2 h postprandially (**a**). The mean glucose (**b**), coefficient of variation of glucose (CV; **c**), and area under the glucose curve (AUC; **d**) during the 2-h postprandial period were calculated. One participant’s data was lost in trials A and C due to the failure of the CGM sensor. The missing data was inserted using regression from the remaining 15 participants’ observations. Two-way ANOVA showed a significant main effect of time (*P* < 0.001) and a time × trial interaction (*P* < 0.001) for glucose time-course responses to meal ingestion (**a**). One-way ANOVA showed a main effect of trial for mean glucose (*P* < 0.01; **b**), and post hoc tests found significant differences between immediate post-meal exercise and control (*P* = 0.004) and pre-meal exercise (*P* = 0.002). There was also a main effect of trial for CV (*P* = 0.03; **c**), with post hoc tests revealing that immediate post-meal exercise was significantly different to control (*P* = 0.02). A main effect of trial was also found for AUC (*P* = 0.005; **d**), and post hoc tests revealed significant differences between immediate post-meal exercise and control (*P* = 0.004) and pre-meal exercise (*P* = 0.002). Data points represent mean ± SD from *N* = 16

All participants completed 3 sets of 10 reps of each exercise and took 7.1 ± 1.8 min to complete each session. The duration of exercise was not different between trials (*P* = 0.21). Mean RPE (11 ± 2 au) during sessions was also not different between trials (*P* = 0.32). Total energy expenditure during each bodyweight exercise session was 53 ± 19 kcal, and RER was 1.04 ± 0.09 au. Figure 4a shows the postprandial interstitial glucose time-course responses to meal ingestion. Mean glucose in the immediate post-meal bodyweight exercise trial was significantly different to control (*P* = 0.004; Fig. 4b) and pre-meal exercise (*P* = 0.002; Fig. 4b). Glucose CV was significantly different between the immediate post-meal bodyweight exercise trial and control trials (*P* = 0.02; Fig. 4c), while glucose AUC in the immediate post-meal exercise trial was significantly different to the control (*P* = 0.004; Fig. 4d) and pre-meal exercise trials (*P* = 0.002; Fig.4d). Measures of daily physical activity and daily energy intake were not different between trials (data not presented), except for daily moderate to vigorous activity level which differed between the two post-meal exercise trials (70.3 ± 34 vs. 42.9 ± 33.3 min; *P* < 0.05). However, when used as a covariate, this difference in daily activity level did not influence between-trial differences in glucose concentrations (data not presented).

Because the caloric expense of each of the three types of activity was not planned to be matched, it was not an a priori aim to directly compare the activity types to one another. That said, a collective analysis of postprandial glucose responses in all three studies (three-way ANOVA) revealed a main effect of time (*P* < 0.001) and a time × trial interaction (*P* < 0.001) where immediate post-meal activity was significantly different to all other trials. Supplemental Fig. S1 summarizes the between-study comparisons.

## Discussion

### Summary of findings

The study shows that low- to moderate-intensity activity immediately following breakfast lowers postprandial glucose exposure (mean and AUC) and glucose variability (CV), while pre-breakfast activity or delayed post-breakfast activity does not. These are valid CGM-derived indices of glucose control according to the international consensus of 2017 [13]. Since postprandial hyperglycemia contributes to elevated HbA1c [31] and cardiovascular disease risk [6, 10] in people with and without diabetes, simple activities like standing, walking, or bodyweight exercises after breakfast may also reduce disease risk. Choosing more vigorous activities may have conferred greater benefit to glucose control. For example, studies using high-intensity intervals or weight lifting show benefit when implemented after a meal [23, 41, 43]. However, vigorous intensity is not always feasible at meal times; it often requires specialized equipment, is not desirable for all people, is precluded in the presence of some chronic conditions, and is initially inappropriate for inactive people [20]. Longer duration activity may also have been advantageous as studies of activity lasting for 2 h post-meal show improved glucose control [8]. Yet, for most people, 2 h of post-meal exercise is not feasible. Furthermore, increasing workout intensity and duration also elicits a greater epinephrine response inducing counter-regulatory mechanisms such as glucagon release and increased hepatic glucose output [27, 28, 37], which may elevate blood glucose and glucose variability, or cause hypoglycemia. By performing standing, walking, and bodyweight activities, our results are externally valid to the real world and feasible in many environments of daily living which separates our study from prior work.

In healthy individuals, ingestion of carbohydrate prior to exercise has in some studies blunted improvements in fitness or performance adaptations [21, 35, 40] but, in the context of glucose control, the optimal activity-meal timing is unclear. The earliest work examined 1 h of cycling in 10 men with diabetes [33, 34]. When cycling was completed 2 h after breakfast, blood glucose was reduced, whereas cycling in the fasted state did not alter blood glucose [33]. One hour of postprandial cycling may, however, not be feasible during daily living. Similar to the current study but performed at dinner time, Colberg et al. found that 15–20-min of walking after dinner lowered blood glucose more so than pre-dinner walking in 12 older-aged, obese, men and women [12]. In 2013, a retrospective study reported greater reductions in blood glucose levels when meals were ingested less than 2 h prior to the beginning of 60-min exercise sessions vs. more than 2 h prior, in 15 older aged, obese, men and women [41]. However, this study was also limited by being a retrospective analysis of food/exercise logs, not including a non-activity control group, and lacking post-meal exercise data. A prospective study by the same authors compared the effects of pre- or post-breakfast treadmill walking in 10 older aged, overweight/obese, men and women [43]. Walking in the fed state reduced glucose AUC compared to no-walking, but pre-breakfast walking was more effective at lowering total AUC glucose of other meals during the day. In 2015, Heden et al. reported that a session of weight lifting lowered postprandial hyperglycemia in 13 middle-aged, obese, men and women but that both pre- and post-dinner exercise were equally beneficial when compared to a non-exercise control group [23]. However, such weight lifting protocols may not be feasible during tasks of daily living for all people. On the contrary, Borer and colleagues found that daylong blood glucose levels were lower when 2 h of walking were completed 60 min before a meal rather than 60 min after a meal, in 9 overweight, middle to older aged women [8]. However, this study had no non-active control group and a total of 4 h of walking in 2-h bouts was used, which is an unrealistic dose to incorporate into daily living. The studies summarized above (reviewed in [39]) have diverse study designs, a lack of appropriate controls in some cases, and dichotomous outcomes. Our data adds clarity to the field.

Physical activity is defined as any muscle-induced bodily movement requiring energy expenditure. In 2018, the global prevalence of insufficient physical activity was 27.5% [22]. Disease risks of inactivity are undeniable [7], and the increasing global prevalence of inactivity is not only alarming, but the health consequences of poor glucose control are preventable with current public guidelines [2, 44]. However, public guidelines are often vague and not specific enough for the public to use. Our results showing that light- to moderate-intensity physical activity following a meal lowers postprandial glucose exposure and variability may provide a useful update to public guidelines to further reduce the health risks associated with hyperglycemia. While activities like walking and bodyweight exercises may be expected to improve glucose control [24], surprisingly, even standing up induced some benefit, albeit mild, despite being very light intensity (RPE = 6). Standing was not intended to match the energy expenditure of walking or body weight exercises, but if standing up is a person’s only feasible activity following breakfast, then it is clearly more beneficial than sitting down. The mechanisms of glucose metabolism during standing have not been studied, but lower limb blood flow is increased during standing compared to sitting [4] and electromyograph (EMG) activity of the large muscle groups required for standing is twofold above that when sitting [19]. So, one may speculate that standing increases glucose delivery to muscle tissue during contraction-mediated glucose uptake.

This new work studied postprandial glycemia in the context of breakfast rather than other meals to control for several important variables. First, by using breakfast, the effect of activity on glucose control could be examined in the overnight fasted (trial B) versus fed state (trials C and D) and glucose clearance of the meal was not influenced by the Staub-Traugott repeated-meal effect [1]. Second, skeletal muscle metabolism in humans follows a diurnal pattern under the control of clock genes [29], so circadian rhythm likely influences the interplay between activity-meal timing and postprandial glucose control. Visual analogue scale questionnaires evaluating gastrointestinal sensations also indicated that feelings of hunger were lowest and feelings of fullness were highest when activity was after breakfast rather than before (Supplemental Fig. S1). The relevance of this to long-term appetite control and energy balance is unknown. Of note, however, is that bodyweight exercise immediately after breakfast increased feelings of nausea. Nonetheless, bodyweight exercises were also a time-efficient (~ 7 min/session) means of improving postprandial glucose control in comparison to other activities (Supplemental Fig. S2). Therefore, the best physical activity for glucose control is dependent on people’s environment, time constraints, and gastrointestinal responses.

### Limitations

Because intestinal absorption of liquid meals is faster than solid meals, the appearance of blood glucose after meal ingestion may be slower if solid meals were used. It would be prudent, therefore, for future work to examine the influence of different exercise-meal timings when using solid foods. Furthermore, since CGM was used, the data show glucose levels in interstitial fluid (ISF) not blood, and while the two are correlated, there is a time-delay in glucose changes between blood and the ISF. Also, since nondiabetic individuals were included, the findings should be extrapolated to diabetes patients with care. Nonetheless, ~ 90% of the world’s population are non-diabetic, so these findings have great impact on informing public health policy for optimizing postprandial glucose control. Another important consideration is that the types and/or duration of the physical activities used, while generally feasible, may be difficult to implement in some work environments at all times of the day. While this new data advances scientific knowledge on the acute responses to exercise and nutrient timing, it does not address longer-term adaptation. Both acute responses and chronic adaptations are important, since they do not necessarily respond in the same way. For example, with high-intensity vigorous exercise, van Proeyen et al. (2010) found that long-term adaptations to post-meal exercise had less impressive effects on glycemic control than pre-meal (fasted) exercise during a 6-week hypercaloric fat-rich diet [35]. Lastly, our study design aimed to look at three different activity types on meal timing and activity rather than comparing which activity type is ideal, which to do so would require impossibly matching of energy expenditure between standing, walking, and body exercises, as well as 16 trials per participant. Despite some limitations, this is the first randomized controlled trial to identify the optimal meal-activity timing that best improves blood glucose control while examining types of activity that are simple to integrate into habits of daily living.

### Conclusion

A low- to moderate-intensity physical activity that raises energy expenditure above resting levels is best implemented immediately after breakfast to elicit the best postprandial glucose control. This finding should prompt an update to current physical activity guidelines [2, 20, 44]. Given the distinct influence of activity-meal timing on blood glucose control following breakfast, the large inter-individual heterogeneity in the therapeutic effect of exercise (reviewed in [38] and the lack of improvement in blood glucose control in some long-term exercise studies [9, 14, 26, 42]) highlights the importance of considering exercise-meal timing in the experimental design of clinical trials. Indeed, as of 2019, no long-term randomized, controlled exercise intervention study with a primary focus on glucose control has reported activity-meal timing or indicated whether exercise sessions were conducted in the fed or fasted state. Based on the results of the current study, a prospective, long-term randomized controlled trial that determines the effect of daily post-meal physical activity on cardiometabolic risk and/or mortality is warranted.

## Electronic supplementary material

ESM 1 (DOCX 473 kb)

ESM 2 (DOCX 527 kb)

ESM 3 (DOC 219 kb)
