# Supplementary material for: Immediate post-breakfast physical activity improves interstitial postprandial glycemia: a comparison of different activity-meal timings
Source: Pflugers Arch. 2019 Aug 8;472(2):271–80. doi: 10.1007/s00424-019-02300-4 (PMC7035221; doi:10.1007/s00424-019-02300-4)

**Supplemental Figure S1. Feelings of hunger, fullness, and nausea.**

Visual analogue scale questionnaires to assess gastrointestinal feelings were administered in each trial immediately before and immediately after each activity. VAS questionnaires being inadequately completed in two trials in the standing and walking studies so those data represent mean ± SD for N=14. In the bodyweight exercise study, three participants inadequately completed their questionnaires so those data represent mean ± SD for N=13.

During standing, hunger was lower in both post-meal standing trials compared to pre-meal standing (both P<0.001) and control (both P<0.05; **Panel A**). There was no pre vs. post-standing difference in hunger. Fullness was lower in pre-meal standing but higher in delayed post-meal standing compared to control (both P<0.05); fullness in both post-meal standing trials was also greater than in pre-meal standing (both P<0.001; **Panel D**). There was no pre vs. post-standing difference in fullness. Nausea was unaffected by trial or pre/post-standing (**Panel G**).

During walking, hunger was lower in both post-meal walking trials compared to pre-meal walking compared to (both P<0.05; **Panel B**). For hunger, there was no pre vs. post-walking difference. Fullness was greater in delayed post-meal walking than in control and pre-meal walking (P<0.05 and P=0.002, respectively) while fullness in immediate post-meal walking was also greater than in pre-meal walking (P<0.05; **Panel E**). There was no pre vs. post-walking difference in fullness, and nausea was unaffected by trial or pre/post-walking (**Panel H**).

During the bodyweight exercises, hunger was lower in the post-meal exercise trials compared to pre-meal exercise (both P<0.001) and control (P=0.001 and P=0.01; **Panel C**). In immediate post-meal exercise, hunger was also lower after exercise compared to before-exercise (P<0.001). Fullness was lower in pre-meal exercise compared to the post-meal exercise trials (P=0.003 and P<0.05, respectively), and fullness was increased following exercise compare to before-exercise in the post-meal exercise trials (P<0.001 and P=0.003, respectively; **Panel F**). Nausea was greater in immediate post-meal exercise compared to control, pre-meal exercise, and delayed post-meal exercise (P=0.005, P<0.001, P=0.003, respectively; **Panel I**). Nausea was also increased by exercise in immediate post-meal exercise only (P<0.001).


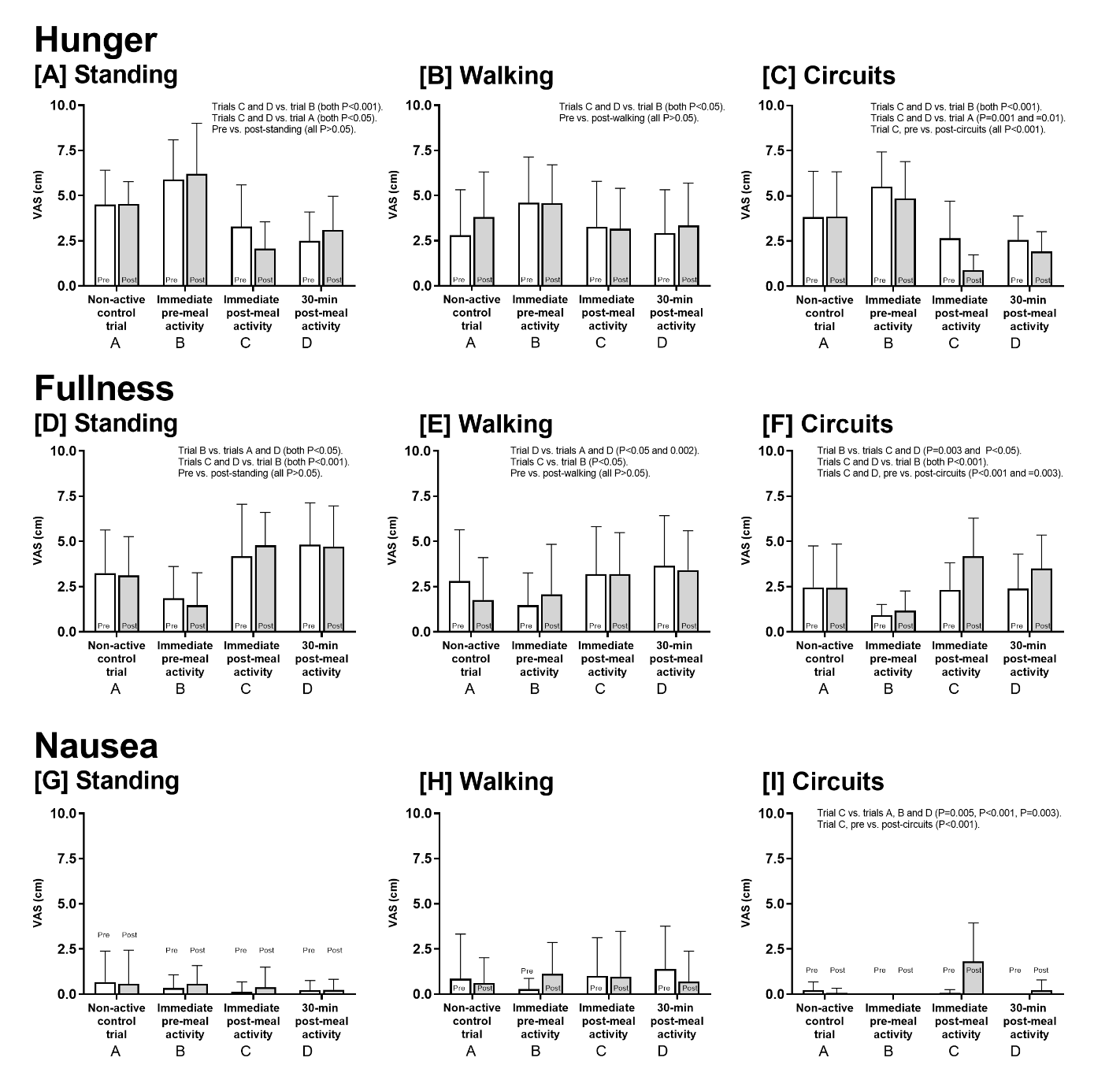

Supplement: Supplementary file 1 — (DOCX 473 kb) [file 424_2019_2300_MOESM1_ESM.docx]
