# Supplementary material for: Immediate post-breakfast physical activity improves interstitial postprandial glycemia: a comparison of different activity-meal timings
Source: Pflugers Arch. 2019 Aug 8;472(2):271–80. doi: 10.1007/s00424-019-02300-4 (PMC7035221; doi:10.1007/s00424-019-02300-4)

**Supplemental Figure S2. Comparison of glycemic control between studies.**

Because the dose of exercise (total energy expended) was different in each study’s physical activity modality, direct statistical comparisons of the effects of standing vs. walking vs. bodyweight exercises are not appropriate. However, there is clinical relevance to viewing the comparisons. Therefore, the mean, coefficient of variance, and area under the curve values can be visually compared in this figure. The dotted line helps compare means to trial A in the standing study. Data represent mean ± SD.


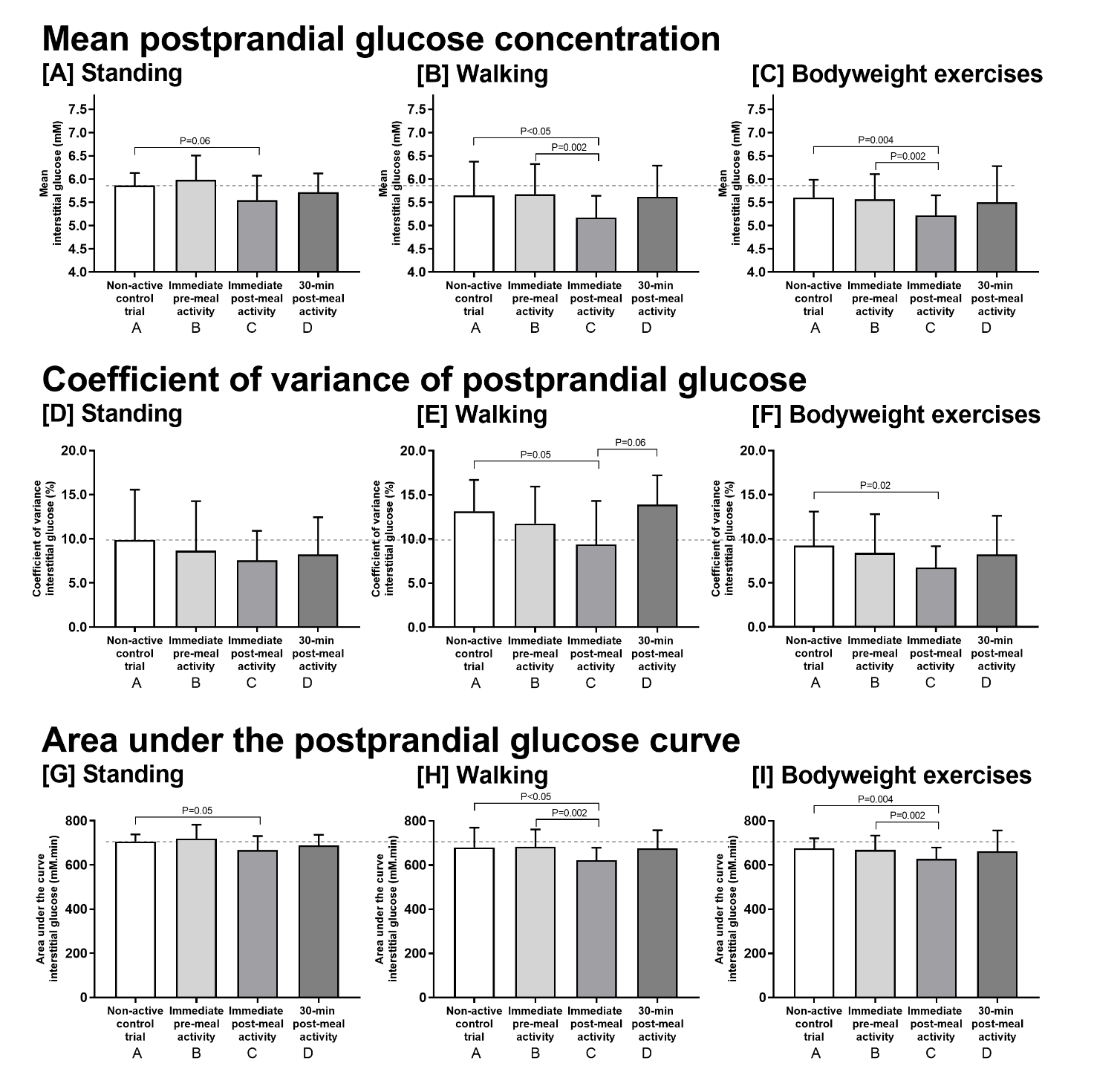

Supplement: Supplementary file 2 — (DOCX 527 kb) [file 424_2019_2300_MOESM2_ESM.docx]
